# Lignans in Spirits: Chemical Diversity, Quantification, and Sensory Impact of (±)-Lyoniresinol

**DOI:** 10.3390/molecules24010117

**Published:** 2018-12-30

**Authors:** Delphine Winstel, Axel Marchal

**Affiliations:** Unité de recherche Œnologie, EA 4577, USC 1366 INRA, Université de Bordeaux, ISVV, 33882 Villenave d’Ornon, France; delphine.winstel@u-bordeaux.fr

**Keywords:** Lignan, bitterness, taste-active compound, quantification, oak ageing

## Abstract

During barrel aging, spirits undergo organoleptic changes caused by the release of aroma and taste compounds. Recently, studies have revealed the bitter properties of oak wood lignans, such as (±)-lyoniresinol, and their contribution to wine taste. To evaluate the impact of lignans in spirits, a targeted screening of 11 compounds was set up and served to validate their presence in this matrix, implying their release by oak wood during aging. After development and validation of a quantification method, the most abundant and the bitterest lignan, (±)-lyoniresinol, was assayed by liquid chromatography–high resolution mass spectrometry (LC-HRMS) in spirits. Its gustatory detection threshold was established at 2.6 mg/L in spirits. A large number of samples quantified were above this detection threshold, which suggests its effect of increased bitterness in spirit taste. Significant variations were observed in commercial spirits, with concentrations ranging from 0.2 to 11.8 mg/L, which could be related to differences in barrel aging processes. In “eaux-de-vie” of cognac, concentrations of (±)-lyoniresinol were observed in the range from 1.6 mg/L to 12 mg/L. Lower concentrations were measured for older vintages.

## 1. Introduction

Spirits are alcoholic beverages, traditionally consumed for human enjoyment. Their sensory quality is strongly influenced by their different production stages [1]. First, the elaboration of spirits needs a raw material with a high content of sugars naturally present in fruits (apple, pear, or grape) for calvados, cognac, armagnac, and brandy; of carbohydrates and starch (corn, barley, or rye) for bourbon and whiskey; or of sugarcane for rum. Thereafter, yeast fermentation, distillation techniques in pots or column stills, maturation in oak barrels, and bottling play their own special part in aroma and taste formation [2,3]. From a chemical point of view, spirits are a highly complex matrix characterized by an ethanol concentration that is usually between 36% to 55% *v*/*v* and a high content of volatile and nonvolatile compounds.

With the development of sensitive and resolutive analytical techniques, such as GC-olfactometry and GC-MS, hundreds of volatile components have been identified in spirits over the last few decades [3,4,5,6,7]. These compounds are mainly esters, alcohols, aldehydes, and volatile acids coming from grapes or formed during fermentation. 

Contrary to volatile compounds, nonvolatile molecules present in spirits are exclusively acquired after distillation. Most of them are released from oak wood during aging. Indeed, barrel aging is a crucial step during which the organoleptic properties of spirits are fine-tuned [8]. The color, structure, aroma, and taste of spirits are modified during oak aging [9,10,11,12], and it is commonly acknowledged that the overall quality improves. While the key aromatic compounds released from oak wood in spirits are well known [13,14], modifications to gustatory properties during this process have been only partially explained. Recent studies have demonstrated the influence of oak wood on the softening of wine and spirits with the discovery of natural sweet compounds called quercotriterpenosides [15,16]. Furthermore, the astringency and bitterness of ellagitannins, a major class of oak wood compounds, have also been studied. Glabasnia and Hofmann showed that the detection thresholds (DTs) of the main hydrolysable tannins were significantly higher than their concentrations in wines, suggesting their low influence on wine bitterness [17]. So far, no correlation between bitterness and oak tannins has been clearly established [18]. Moreover, a large number of studies have examined the phenolic composition of spirits. Mainly nonflavonoids have been identified and quantified in aged spirits, most of them of low molecular weight [19,20]. Phenolic acids such as gallic acid are the most abundant phenolic compounds in spirits, followed by phenolic aldehydes (vanillin, sinapaldehyde, syringaldehyde), lignans, phenyl ketones, and coumarins such as scopoletin [21,22,23,24,25].

Among these nonvolatile compounds present in spirits, lignans appear to be particularly interesting, since previous studies have demonstrated the bitter properties of lyoniresinol [16] and various derivatives. Lyoniresinol has been described as the most abundant lignin in sessile oak, and its detection threshold has been established at 1.5 mg/L in wine [26]. A complementary study showed that only (+)-lyoniresinol was bitter, with a detection threshold established at 0.5 mg/L in wines [27]. This enantiomer was quantified in various wines at concentrations higher than this value, demonstrating its contribution to wine bitterness [28]. Yet the sensory properties of a molecule, and in particular its detection threshold, can be strongly influenced by the nature of the matrix from an olfactory or gustatory point of view [29,30,31,32]. Consequently, the contribution of lignans to spirit taste cannot be presumed from the results obtained in wine. A better knowledge on oak wood and spirits composition appears particularly interesting to improve the quality of these products with high economic interest.

Based on these observations, the present work aimed at studying the occurrence of oak wood lignans in spirits and their taste contribution. First, samples of commercial spirits were screened by liquid chromatography–high resolution mass spectrometry (LC-HRMS) to search for the presence of targeted oak wood lignans. Considering the strong bitterness induced by (±)-lyoniresinol, the detection threshold of this lignan was established in a spirit matrix. Finally, after development and validation of an LC-HRMS method, a racemic mixture of lyoniresinol was quantified in various samples of commercial and experimental spirits in order to investigate its sensory impact along with the influence of oenological parameters on its content.

## 2. Results and Discussion

### 2.1. Chemical Diversity of Oak Lignans in Spirits

**Figure 1:**
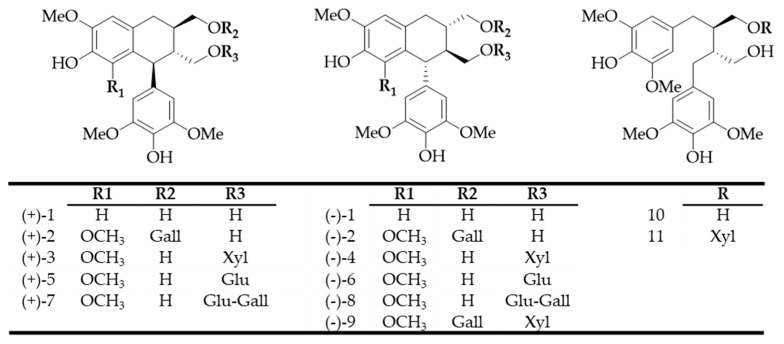
Chemical structures of lignans **1**–**11**. Xyl, Glu, and Gall correspond, respectively, to β-xylopyranose, β-glucopyranose, and galloyl.

Previous studies have focused on the diversity and gustatory importance of lignans present in oak wood [33]. Using an LC-HRMS guided purification protocol, 11 lignans (Compounds **1**–**11**, Figure 1), natural derivatives of lyoniresinol, were isolated and identified from an oak wood extract [26,34]. In addition, it has been proven that all these molecules are released in wine aged in oak barrels. In this matrix, some of the galloyl, glucosyl, and xylosyl derivatives have been described as bitter but with a lower intensity than lyoniresinol [26].

**Figure 2:**
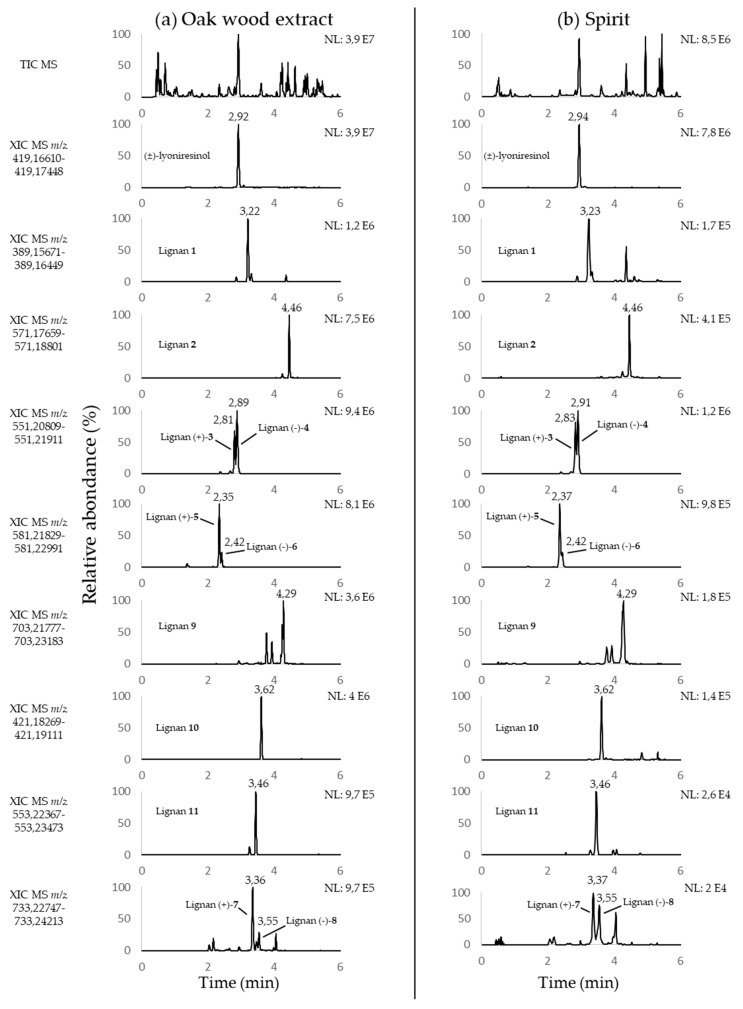
Negative LC-ESI-FTMS XIC of an (**a**) oak wood extract and (**b**) a spirit corresponding to [M − H]^−^ ions of lyoniresinol and lignans **1**–**11** (from top to bottom).

Their presence has not been reported in spirits until now, even though maturation in barrels is crucial for fine-tuning their sensory properties. To determine whether these lignans were likely to impact the taste of spirits, their presence was first investigated by screening a cognac aged for 23 years. Based on mass measurement accuracy, LC-HRMS is a powerful technique in screening samples by targeting the characteristic *m/z* ions of specific empirical formulas. To this end, the chromatographic and spectrometric conditions described by Marchal et al. [26] were applied. Extracted ion chromatograms (XICs) were obtained in an oak wood extract (Figure 2a) and in a spirit (cognac, Figure 2b) by considering the *m/z* ratios specific to the deprotonated ions ([M − H]^−^) of (±)-lyoniresinol and lignans **1** to **11** with a 3-ppm tolerance window (Figure 2).

Similar signals were detected in XICs of both samples. Moreover, analysis in higher energy collision dissociation (HCD) fragmentation mode revealed the same main fragment ions in the two matrices. Concomitantly with the specificity of mass measurement (<3 ppm) and retention time similarity (<0.02 min), these results demonstrated that lignans **1**–**11** were present in the analyzed spirits. The presence of lyoniresinol had already been established in oak wood extracts [35,36], wines [21], and spirits [8,22,35,37]. Lignans **7** and **8** had also been identified in these three matrices [34]. However, lignans **1**–**6** and **9**–**11** had been described in wines [26,34], but never in spirits. A comparison of the signal intensity of all the lignans suggested that (±)-lyoniresinol might be the most abundant of them in spirits. These results confirmed recent studies, in which the combination of sensory analysis and quantitative studies established that lyoniresinol is both the most abundant lignan released from oak wood in wine and one of the bitterest.

The detection threshold of a compound is the concentration beyond which this molecule is perceived by one half of a panel. In oenology, detection thresholds have been measured, mainly on olfactory compounds, in different matrices such as water, wine, or spirits [29,30,32]. These studies have shown that the nature of the matrix can have a significant impact on the olfactory properties of a compound. In the same way, taste-active compounds can also be strongly affected by the matrix. For instance, the detection threshold was calculated for a bitter compound, caffeine, in water and liquid food at 94 and 184 mg/kg, respectively [31]. These results underline the importance of calculating a detection threshold of (±)-lyoniresinol in a spirit matrix to determine its effective impact on spirit taste.

### 2.2. Sensory Impact and Quantification of (±)-Lyoniresinol in Spirits

Previous studies have demonstrated that lyoniresinol significantly contributes to the bitterness of oaked wines [26]. However, depending on the studied matrix, the sensory properties of lyoniresinol can change. For this reason, the detection threshold must be evaluated in “eau-de-vie” of cognac and compared to quantitative values measured in spirits by LC-HRMS.

#### 2.2.1. Determination of (±)-Lyoniresinol Detection Threshold in Spirits

The gustatory impact of (±)-lyoniresinol had been previously established in a white wine by Marchal et al. [26]. The group taste threshold was calculated to be 1.5 mg/L, with a wide range of individual detection thresholds from 0.125 to 11.3 mg/L. Furthermore, Cretin et al. demonstrated that only (+)-lyoniresinol exhibited a bitter taste compared to its enantiomer, described as tasteless or slightly sweet [27,28]. As not enough (+)-lyoniresinol was available in our laboratory to determine its detection threshold, the sensory studies presented in this work were carried out with a racemic mixture, which is naturally present in oak wood.

The detection threshold of lyoniresinol was determined using the 3AFC (three-alternative forced choice) method [38,39]. Solutions of (±)-lyoniresinol at various concentrations were prepared according to a geometric progression with a ratio of 2, and presented in 3AFC tests. Two sessions were organized to avoid excessive tiredness among the tasters. The (±)-lyoniresinol group threshold was established at 2.6 mg/L with strong inter-individual variability. Indeed, individual detection thresholds covered a range from 0.35 mg/L to 32 mg/L. The same trends had been described in wine, but the gustatory threshold values were significantly higher in “eau-de-vie”. These variations could be partly due to the higher level of ethanol in the matrix. They confirmed the results of a preliminary study on “eau-de-vie” of armagnac [35].

#### 2.2.2. Development of an LC-HRMS Method to Quantitate Lyoniresinol in Spirits

Previous studies have shown the relevance of using LC-HRMS to quantify (±)-lyoniresinol in spirits, wines, and oak wood macerates [26,28]. The same chromatographic conditions were used for (±)-lyoniresinol quantification in spirits.

The LC-HRMS method previously described for quantification in wine involved an ionization in negative mode [26]. However, preliminary tests were carried out in the same conditions and showed insufficient results regarding linearity and accuracy with the spirit samples. Consequently, a quantification method based on a positive ionization mode was developed for this matrix. LC-HRMS quantification was performed in full scan mode, the selectivity of the detection being ensured by the mass accuracy measurement of the Orbitrap analyzer (<3 ppm) and the repeatability of the retention time.

**Figure 3:**
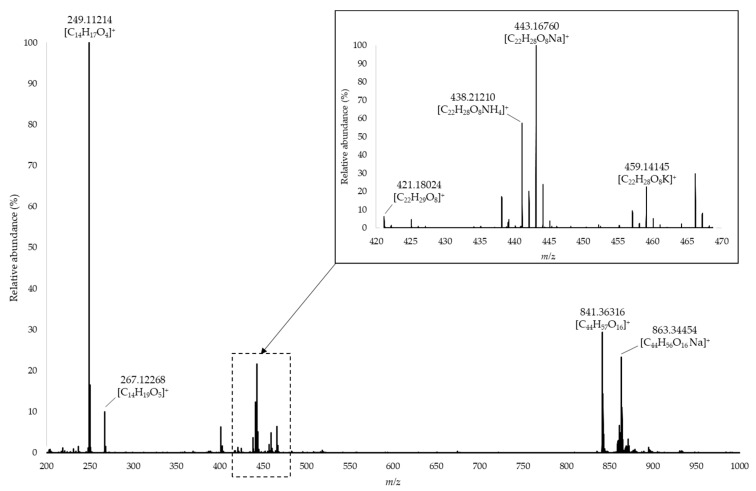
High resolution mass spectrometry (HRMS) spectrum of (±)-lyoniresinol in positive ionization mode.

The full scan HRMS spectrum of (±)-lyoniresinol (C_22_H_28_O_8_) presented several ions corresponding to adducts ([M + Na]^+^, [M + K]^+^, [M + NH_4_]^+^); pseudodimers ([2M + H]^+^, [2M + Na]^+^); and fragments (C_14_H_17_O_4_^+^, C_14_H_19_O_5_^+^) (Figure 3), but the signal corresponding to protonated ion (C_22_H_29_O_8_^+^) was very low, whatever the ionization parameters. Preliminary tests showed that quantification was more reliable by using the fragment ion at *m*/*z* 249.11214 (C_14_H_17_O_4_^+^), which was the most intense signal. This chemical species might result from a loss of a dimethoxyphenol group (C_8_H_10_O_3_) jointly with dehydration. Such fragmentation reactions are well known for lignin derivatives [40] and can be caused by high temperature [41]. Spectrometry parameters were tuned to optimize the response of the *m*/*z* 249.11214 ion.

Absolute quantification was carried out by preparing calibration solutions of pure lyoniresinol in a model solution at 8% *v*/*v*. Orbitrap analysis afforded high accuracy of mass measurement, so extracted ion chromatograms were built with a 3-ppm window around the theoretical *m*/*z* of the C_14_H_17_O_4_^+^ ion. Injection of pure lyoniresinol indicated a characteristic retention time of 2.90 min. This t_R_ was considered for automatic integration of the XIC.

##### Sensitivity

Given the high selectivity of the mass measurement, the notion of signal-to-noise is not suitable for this technique. The detection limit of a molecule is defined as the lowest concentration of this molecule for which a reliable and reproducible signal is observed. In addition, the signal must be different from a blank made under the same conditions. In this study, the method described by De Paepe et al. [42] was used. The lowest levels of the calibration curve (from 2 to 50 µg/L) were injected into five replicates. Precision (relative standard deviation (RSD)%) and accuracy (recovery of back-calculated concentrations) were obtained for each concentration. The instrumental detection limit (IDL) was defined as the standard deviation at the lowest concentration that could be measured with a precision lower than, e.g., 10%, and an accuracy higher than, e.g., 90%. With this method, it was evaluated at 5 µg/L in spirits. The instrumental quantification limit (IQL) was defined as twice the corresponding IDL (10 µg/L). Limits of detection (LOD) and quantification (LOQ), reassessed using the dilution factor, were calculated at 25 µg/L and 50 µg/L, respectively.

##### Linearity and Accuracy

The working range was chosen taking into account the IQL previously determined. A quadratic calibration curve (1/*x* statistical weight) was obtained with a good correlation coefficient (*R*² of 0.9999) for a range from 10 µg/L to 10 mg/L. The recovery of back-calculated concentrations was higher than 90% at each method calibration level, establishing the accuracy. 

##### Specificity

Specificity was assessed by mass accuracy and the repeatability of retention times. These parameters were checked for each injection of calibration solutions and samples. Low variations in retention time (<0.04 min) and a mass deviation lower than 2.2 ppm between experimental and theoretical values were observed for lyoniresinol, guaranteeing the specificity of the method. Moreover, no signal was detected in non-oaked “eau-de-vie”.

##### Repeatability and Trueness

To determine intraday repeatability (RSD%), five replicates of two concentrations (100 µg/L and 1 mg/L) of the calibration curve were successively injected. Values lower than 4% were obtained for both concentrations, guaranteeing the repeatability of the method.

Trueness was determined by calculating the recovery ratios of three different samples of cognac spiked with stock solutions for additions of 100 µg/L and 1 and 2 mg/L. These recovery ratios ranged from 85% to 98%. These slight variations could be explained by the highly complex matrix of spirits. However, the results remained in accordance with common specifications [43] and established the trueness of the method. Interday repeatability was estimated by injections of the same standard solutions for five successive days. As usually observed for LC-ESI-MS analysis, the RSD values were quite high. To overcome this issue, all the calibration solutions were injected for each quantitative analysis of an unknown sample.

All of the results proved the ability of the LC-HRMS method to quantify lyoniresinol in spirits (Table 1).

#### 2.2.3. Application of the Method to Quantitate Lyoniresinol in Spirits

##### Content of Lyoniresinol in Various Commercial Spirits

**Figure 4:**
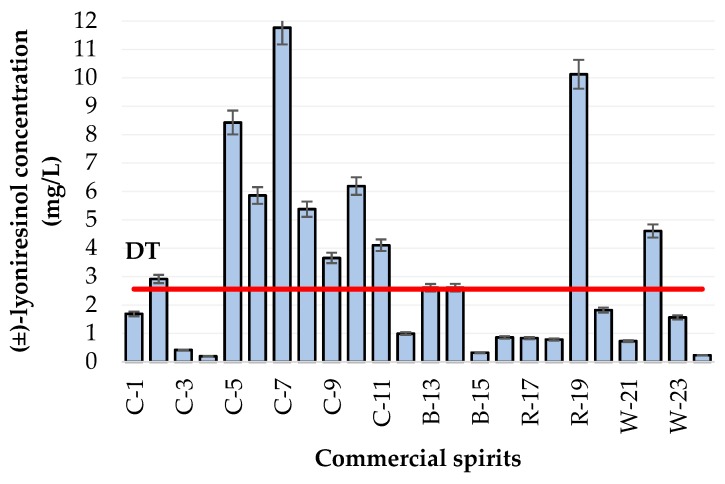
Variations in (±)-lyoniresinol content in 24 commercial spirits. The red line represents the level of the gustatory detection threshold (DT).

Twenty-four commercial spirits were analyzed to assess the range of lyoniresinol amounts in some cognacs, but also whiskies, rums, and other brandies using the LC-HRMS method previously validated. Lyoniresinol was detected in all samples, at concentrations ranging from 0.2 to 11.8 mg/L with a mean value of 3.3 mg/L. The results are illustrated in Figure 4. There were not enough samples to carry out a statistical study, but among the spirits richest in lyoniresinol, there was a majority of cognacs (C-2 and C-5 to C-11), as well as two brandies (B-13 and B-14), one rum (R-19), and one whiskey (W-22). A comparison to sensory data highlighted that the lyoniresinol concentration was above the detection threshold in these 12 spirits, establishing the sensory relevance of this compound, which is likely to contribute to the bitterness of oaked spirits. 

Furthermore, the significant variations in lyoniresinol observed between these commercial spirits could have been due to various factors, and some hypotheses can be evoked. First, the analyzed commercial spirits were aged in contact with oak wood, so these observations could have been related to aging conditions. The influence of the aging container on the lyoniresinol content of a white wine has already been established, confirming that lyoniresinol is released from oak wood to wine [26]. Additionally, this study showed that new oak barrels contained more lyoniresinol. Thus, the variations observed in the analyzed spirits could have been due to the proportion of new oak barrels used during the aging of the spirits. Previous studies have also demonstrated that the concentrations of molecules vary according to the use of new or used barrels, barrels previously used in a maturation cycle [9]. Indeed, Piggott et al. noted differences in the levels of phenolic compounds depending on three types of casks. The study showed an increase in concentrations of nonvolatile compounds, such as vanillin, syringaldehyde, and syringic acid, as well as a reduction in coniferaldehyde and sinapaldehyde concentrations in 36-month-old whiskey distillates [10]. As spirits are aged for a longer period than wine [8], aging time and aging container could explain the significant variations in lyoniresinol observed in these samples, but such information was not available for the commercial spirits analyzed in this study. The potential influence of aging conditions on lyoniresinol concentrations in spirits needs to be clarified in further studies.

Second, various cooperage parameters could have affected the chemical composition of oak wood. Previous works have shown that the concentration of oak molecules released in wine or spirits could vary according to the botanical species or geographical origin of the oak [44,45,46]. However, Cretin et al. demonstrated that there was no significant effect of oak species on lyoniresinol content [28]. 

In addition, during barrel making, the wood undergoes a series of stages that influence its oenological quality, most notably seasoning and toasting of the staves. These technological features affect the structure and the chemical composition of the wood, but also the future matrix with which it will be in contact [47,48,49,50,51]. Indeed, Cretin et al. studied the influence of wood toasting temperature on lyoniresinol and showed that this compound was slightly degraded at around 250 °C [28], in line with another study [52].

##### Content of Lyoniresinol in Various Vintages of the Same Spirits

**Figure 5:**
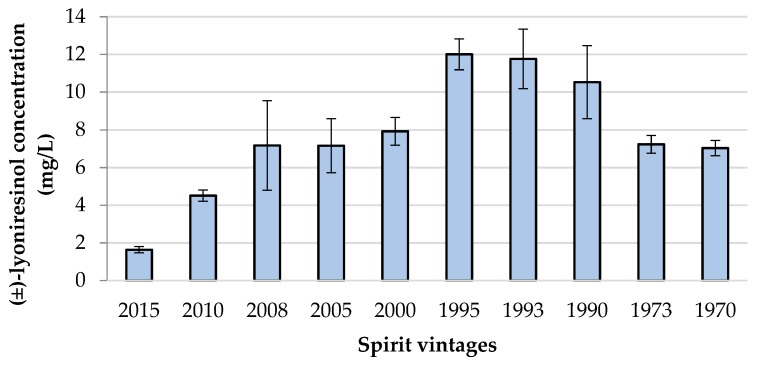
Concentrations of (±)-lyoniresinol in 10 vintages of cognac “eaux-de-vie” coming from the same distillery.

Lyoniresinol was quantified in a series of “eaux-de-vie” of cognac of 10 different vintages from the same distillery and using similar aging conditions. The samples were not commercial cognac, but “eaux-de-vie” still undergoes the aging process in barrels. They were matured in used barrels (a 350-L coarse grain oak barrel). For each vintage, a sample was collected from five different barrels and analyzed. The concentrations presented in Figure 5 correspond to the mean values of these five replicates. The measured values ranged from 1.6 mg/L (2015) to 12 mg/L (1995). For each vintage, the coefficient of variation between the five replicates was relatively low (from 5.7% to 33.2%), revealing a good homogeneity between barrels. From 2015 to 1995, the results showed that the older the “eau-de-vie”, the higher the level of lyoniresinol, confirming previous observations [27,35,37]. Conversely, for older vintages, lower concentrations were measured. This could suggest a degradation of lyoniresinol in long-time barrel storage. Furthermore, aging in used barrels implies a lower extractable potential of the compounds than in new oak barrels and can influence the lyoniresinol content in these vintages. However, this hypothesis needs to be studied more deeply, since the results could also have been due to modifications to aging practices in the distillery or changes in barrel supplies.

Despite the release of lyoniresinol, a bitter compound, spirits are known to improve during oak wood aging. Research has highlighted the impact of other taste-active compounds such as quercotriterpenosides [16], which could be released at the same time from oak wood and might modulate the effect of lyoniresinol on the taste balance in spirits.

## 3. Materials and Methods

### 3.1. Chemicals

d-(+)-glucose, d-(−)-fructose, and quinine sulfate were purchased from Sigma-Aldrich (Saint-Quentin-Fallavier, France). Ultrapure water (Milli-Q purification system, Millipore, France) and HPLC grade solvent (acetonitrile, ethanol, ethyl acetate, *n*-heptane, methanol, and propan-2-ol; VWR International, Pessac, France) were used for sample preparation and lignan purification. Acetonitrile (ACN) and water used for chromatographic separation were LC-MS grade and were purchased from Fisher Chemical (Illkirch, France). Lignans were isolated as previously described by Marchal et al. [26].

### 3.2. LC Analysis

The HPLC appliance consisted of an HTC PAL autosampler (CTC Analytics AG, Zwingen, Switzerland) and an Accela U-HPLC system with quaternary pumps. For (±)-lyoniresinol quantitation, a C18 column (Hypersil Gold 2.1 × 100 mm, 1.9-μm particle size, Thermo Fisher Scientific) was used with water (Eluent A) and ACN (Eluent B) as mobile phases. The flow rate was set at 600 μL/min, and the injection volume was 5 μL. Eluent B varied as follows: 0 min, 14%; 0.5 min, 14%; 1.5 min, 19%; 2 min, 19%; 4.5 min, 38%; 4.6 min, 98%; 6.9 min, 98%; 7 min, 14%; 8.6 min, 14%. 

### 3.3. HRMS 

An Exactive Orbitrap mass spectrometer equipped with a heated electrospray ionization (HESI II) probe (both from Thermo Fisher Scientific, Les Ulis, France) was used. The mass analyzer was calibrated each week using Pierce^®^ESI Negative and Positive Ion Calibration solutions (Thermo Fisher Scientific).

#### 3.3.1. Screening

To perform targeted screening, the ionization and spectrometric parameters, optimized in negative mode, were previously described by Marchal et al. [26]. Table 2 summarizes all the data. 

#### 3.3.2. Quantification 

For quantitation of (±)-lyoniresinol in spirits, mass acquisitions were performed and optimized in positive Fourier transform (FTMS) ionization mode. The ionization and spectrometric parameters are described in Table 3. All data were processed using the Qual Browser and Quan Browser applications of Xcalibur version 3.0 (Thermo Fisher Scientific) [45].

### 3.4. Spirits and Sample Preparation

Two series of spirits were used in this study. Lyoniresinol quantitation was assessed in 24 commercial spirits (including 12 cognacs, 4 grape brandies, 3 rums, 4 whiskies, and 1 bourbon). All of these were aged in oak wood. Table 3 summarizes all the features of these spirits, which were randomly chosen among spirits commercially available and well distributed in France.

The second set of spirits, supplied by Rémy-Martin, consisted of 10 vintages from 1970 to 2015, with five replicates for each year. The samples came from the same distillery and used similar aging conditions. 

All concentrations were expressed in mg/L of spirits. 

A spirit is a matrix with a high alcohol content, so a dilution is necessary before any injection. This prevents deterioration of the chromatographic separation and allows all concentrations to be included in the working range. For quantitative analysis, the percentage of alcohol ranged from 71 to 38% *v*/*v*, and the spirit samples were reduced to 8% alcohol with water and 0.45 µm filtered. The diluted spirits were injected directly into LC-HRMS using the chromatographic and spectrometric parameters described above.

### 3.5. Preparation of Calibration Solution for Lignan Quantitation

A stock solution of (±)-lyoniresinol (1 g/L) was prepared in ethanol. One range of calibration was prepared by successive dilution of this solution in hydroethanolic solution (8%, *v*/*v*) in order to supply calibration samples (10 mg/L, 5 mg/L, 2 mg/L, 1 mg/L, 500 µg/L, 200 µg/L, 100 µg/L, 50 µg/L, 20 µg/L, 10 µg/L, 5 µg/L, 2 µg/L). Detection of (±)-lyoniresinol was based on the theoretical exact mass of the most intense ion, the fragment ion at *m*/*z* 249.11214, and its retention time at 2.90 min. Peak areas were determined by automatic integration of extracted ion chromatograms built in a 3-ppm window around the exact mass of the C_14_H_17_O_4_^+^ ion. 

### 3.6. Method Validation for Quantitation of Lyoniresinol on C18 Column 

The quantitation method of (±)-lyoniresinol in spirits was validated by studying sensitivity, linearity, specificity, intraday repeatability, and trueness. A calibration curve was established by plotting the areas for each concentration level versus the nominal concentration. Quadratic regression was chosen with a 1/*x* statistical weight.

The sensitivity of the LC-HRMS method was determined following the approach described by De Paepe et al. [42]. Linearity was evaluated by correlation coefficient (*R*^2^) and by deviations of each back-calculated standard concentration from the nominal value. To evaluate repeatability, the intraday precision was determined by injecting five replicates of two intermediate calibration solutions (100 and 1000 μg/L), and the relative standard deviation (RSD%) was calculated. Trueness was checked by calculating the recovery ratio (between measured and expected areas) from three samples of spirits (C-7, C-8, EDV-1995). They were chosen among the analyzed samples and were spiked with calibration solution corresponding to an addition of 100 µg/L and 1 and 2 mg/L of (±)-lyoniresinol. Specificity was assessed by evaluating the mass accuracy and retention time repeatability. These parameters were determined concomitantly with the precision and trueness analysis described above.

### 3.7. Sensory Analysis

Tasting sessions took place in a specific air-conditioned room at 20 °C equipped with individual booths and normalized glasses. The “eau-de-vie” used for sensory analysis was a non-oaked spirit adjusted to 40% *v*/*v* of ethanol with pure and demineralized water (eau de source de Montagne, Laqueuille, France). The absence of (±)-lyoniresinol in this matrix was checked by LC-HRMS analysis. 

Results obtained from the sensory tests were statistically interpreted following the norms published by the International Organization for Standardization (ISO) [38]. 

#### 3.7.1. Panel Training

The panel consisted of 24 wine tasters, 7 men and 17 women, aged from 20 to 45 years. The aim of this training session was, first, to accustom the panel to sweet and bitter perceptions (glucose, fructose, and quinine sulfate aqueous solutions), and in the second session to familiarize all panelists with a new matrix with a higher alcohol concentration (40%, *v*/*v*).

First, two aqueous solutions containing, respectively, glucose and fructose at 10 g/L, and quinine sulfate at 20 mg/L, were presented to the tasters to illustrate sweet and bitter tastes. These solutions were made with demineralized water (eau de source de Montagne, Laqueuille, France). 

In a second session, solutions containing these compounds at the same concentrations, but in a non-oaked “eau-de-vie” (40%, *v*/*v*), were presented to the tasters. They were asked to rate the sweetness and bitterness intensities of each solution on a 0–10 scale. The results were interpreted by a one-way analysis of variance (ANOVA). For each parameter, the homogeneity of the variance was assessed using the Levene test. ANOVA’s statistics were considerably below the *p*-value of 0.05. Indeed, results were significant with a *p*-value of 0.0001 for the sweet solution and a *p*-value of 0.003 for the bitter solution. Training showed the reliability of the panel to distinguish sweet and bitter tastes even in a complex matrix.

#### 3.7.2. Determination of Lyoniresinol Taste Threshold in Spirits

The taste threshold of (±)-lyoniresinol was evaluated in a non-oaked “eau-de-vie” adjusted at 40% v/v. Due to the higher alcohol concentration present in this matrix and the remanence of the bitter taste, two different sessions were planned to avoid tiredness among the panelists. In the first session, three concentrations (2, 4, and 8 mg/L) were presented in ascending order. Each concentration was displayed according to the 3AFC (three-alternative forced choice) described by ISO 4120:2007 [38]. Concentrations presented in the second session depended on the results from the first session for each taster. If the panelist had given correct answers, three lower concentrations (0.5, 1, and 2 mg/L) were presented to him or her, following a geometric progression of ratio 2, starting with the lowest. Conversely, tasters who did not give any correct answers during the first session received three higher concentrations (8, 16, and 32 mg/L) in the other session. 

Individual thresholds were estimated as the geometrical mean between the lowest concentration of a continuous series of three correct answers and the concentration just below this level. The group threshold was estimated as the geometrical mean between all the individual thresholds.

## 4. Conclusions

This study focused on the use of analytical and sensory techniques to highlight the presence of lignans in spirits and the importance of lyoniresinol. First, an LC-HRMS targeted screening of spirits aged in barrels revealed the presence of 11 lignans. Next, the study focused on the most abundant and the bitterest lignan, (±)-lyoniresinol, to assess its sensory role in spirits. After development and validation of the LC-HRMS method, a racemic mixture of lyoniresinol was quantified in 24 commercial spirits and 10 different vintages of an “eau-de-vie” of cognac. Results showed that in various spirits, (±)-lyoniresinol was above its detection threshold, which was estimated at 2.6 mg/L. This work revealed that this lignan has a significant impact on the taste balance of spirits, as it increases its bitterness. Additionally, high concentrations of (±)-lyoniresinol (up to 12 mg/L) were observed in an oaked “eau-de-vie” of cognac, and its level was detected above the detection threshold for the considered samples, covering a period of almost 50 years. Furthermore, in the analyzed commercial spirits, significant variations were observed, ranging from 0.2 to 11.8 mg/L, and could be partly explained by differences in aging modalities. A similar analytical strategy could be developed to determine the importance of the dextrorotatory enantiomer of lyoniresinol, which exhibits a strong bitter taste, in brandies. This work brings new insight into and a better understanding of the molecular origin of spirit taste. From a practical point of view, studying the parameters likely to affect the level of (+)-lyoniresinol in oak wood and spirits would open up interesting perspectives for better monitoring of the organoleptic properties of spirits.

## Figures and Tables

**Table 1:** Validation parameters for HRMS quantitation of (±)-lyoniresinol in spirits.

Parameters	Matrix/Spirits			
**Sensitivity**	**IDL (µg/L)**	**IQL (µg/L)**	**LOD (µg/L)**	**LOQ (µg/L)**
	5	10	25	50
**Linearity and Accuracy**	**Working Range**		***R*^2^**	
	10 µg/L–10 mg/L		0.9999	
**Specificity**	**t_R_ variation**		**Mass Accuracy**	
	<0.04 min		<2.2 ppm	
**Repeatability and Trueness**	**Intraday Repeatability**			
	100 µg/L		1 mg/L	
	3.35%		3.43%	
	**Recovery**			
	**Samples**	100 µg/L	1 mg/L	2 mg/L
	**EDV-C7**	94%	91%	98%
	**EDV-C8**	87%	85%	97%
	**EDV-1995**	88%	86%	87%

IDL: Instrumental detection limit; IQL: Instrumental quantification limit; LOD: Limit of detection; LOQ: Limit of quantification; EDV: eau-de-vie.

**Table 2:** Ionization and spectrometric conditions for HRMS analyses.

Mass Spectrometer	Exactive	
Use	LC-MS Screening	LC-MS Quantification
Ionization Mode	Negative	Positive
Sheath gas flow ^a^	75	75
Auxiliary gas flow ^a^	18	20
HESI probe temperature	320 °C	320 °C
Capillary temperature	350 °C	350 °C
Electrospray voltage	−3 kV	3.5 kV
Capillary voltage	−60 V	35 V
Tube lens voltage offset	−135 V	120 V
Skimmer voltage	−26 V	18 V
Mass range (in Th)	200–800	200–800
Resolution ^b^	25 000	25 000
Automatic gain control value	10^6^	10^6^

^a^ Sheath gas and auxiliary gas flows (both nitrogen) are expressed in arbitrary units. ^b^ Resolution *m*/Δ*m*, fwhm at *m*/*z* 200 Th.

**Table 3:** Features of commercial spirits.

Samples	Brands	Type	Origin	ABV (Alcohol by Volume)
**C-1**	Brand A	Cognac	France	40
**C-2**	Brand A	Cognac	France	40
**C-3**	Brand A	Cognac	France	40
**C-4**	Brand A	Cognac	France	40
**C-5**	Brand B	Cognac	France	40
**C-6**	Brand B	Cognac	France	40
**C-7**	Brand C	Cognac	France	40
**C-8**	Brand C	Cognac	France	40
**C-9**	Brand C	Cognac	France	40
**C-10**	Brand D	Cognac	France	40
**C-11**	Brand D	Cognac	France	40
**C-12**	Brand E	Cognac	France	42
**B-13**	Brand F	Brandy	South Africa	38
**B-14**	Brand G	Brandy	France	40
**B-15**	Brand H	Brandy	Germany	38
**B-16**	Brand I	Brandy	France	40
**R-17**	Brand J	Rum	Jamaica	43
**R-18**	Brand K	Rum	Guyana	40
**R-19**	Brand L	Rum	Barbados	43
**W-20**	Brand M	Whisky	Ireland	40
**W-21**	Brand N	Whisky	Scotland	40
**W-22**	Brand O	Whisky	Scotland	40
**W-23**	Brand O	Whisky	Scotland	40
**Bo-24**	Brand P	Bourbon	United States	50
